# Quantitative and clinical impact of MRI-based attenuation correction methods in [^18^F]FDG evaluation of dementia

**DOI:** 10.1186/s13550-019-0553-2

**Published:** 2019-08-24

**Authors:** Silje Kjærnes Øen, Thomas Morten Keil, Erik Magnus Berntsen, Joel Fredrik Aanerud, Thomas Schwarzlmüller, Claes Nøhr Ladefoged, Anna Maria Karlberg, Live Eikenes

**Affiliations:** 1 Department of Circulation and Medical Imaging, Norwegian University of Science and Technology, Postbox 8905, N-7491 Trondheim, Norway; 2 Department of Radiology and Nuclear Medicine, St. Olavs Hospital, Trondheim, Norway; 3 Department of Nuclear Medicine and PET Centre, Aarhus University Hospital, Aarhus, Denmark; 4 Department of Radiology, Haukeland University Hospital, Bergen, Norway; 5 Department of Clinical Medicine, University of Bergen, Bergen, Norway; 6 Department of Clinical Physiology, Nuclear Medicine & PET, Rigshospitalet, University of Copenhagen, Copenhagen, Denmark

**Keywords:** PET/MRI, Attenuation correction, z-scores, dementia

## Abstract

**Background:**

Positron emission tomography/magnetic resonance imaging (PET/MRI) is a promising diagnostic imaging tool for the diagnosis of dementia, as PET can add complementary information to the routine imaging examination with MRI. The purpose of this study was to evaluate the influence of MRI-based attenuation correction (MRAC) on diagnostic assessment of dementia with [^18^F]FDG PET. Quantitative differences in both [^18^F]FDG uptake and z-scores were calculated for three clinically available (DixonNoBone, DixonBone, UTE) and two research MRAC methods (UCL, DeepUTE) compared to CT-based AC (CTAC). Furthermore, diagnoses based on visual evaluations were made by three nuclear medicine physicians and one neuroradiologist (PET_CT_, PET_DeepUTE_, PET_DixonBone_, PET_UTE_, PET_CT_ + MRI, PET_DixonBone_ + MRI). In addition, pons and cerebellum were compared as reference regions for normalization.

**Results:**

The mean absolute difference in z-scores were smallest between MRAC and CTAC with cerebellum as reference region: 0.15 ± 0.11 σ (DeepUTE), 0.15 ± 0.12 σ (UCL), 0.23 ± 0.20 σ (DixonBone), 0.32 ± 0.28 σ (DixonNoBone), and 0.54 ± 0.40 σ (UTE). In the visual evaluation, the diagnoses agreed with PET_CT_ in 74% (PET_DeepUTE_), 67% (PET_DixonBone_), and 70% (PET_UTE_) of the patients, while PET_CT_ + MRI agreed with PET_DixonBone_ + MRI in 89% of the patients.

**Conclusion:**

The MRAC research methods performed close to that of CTAC in the quantitative evaluation of [^18^F]FDG uptake and z-scores. Among the clinically implemented MRAC methods, Dixon_Bone_ should be preferred for diagnostic assessment of dementia with [^18^F]FDG PET/MRI. However, as artifacts occur in Dixon_Bone_ attenuation maps, they must be visually inspected to assure proper quantification.

**Electronic supplementary material:**

The online version of this article (10.1186/s13550-019-0553-2) contains supplementary material, which is available to authorized users.

## Background

Magnetic resonance imaging (MRI) is today the preferred imaging modality in the clinical workup of suspected neurodegenerative disease due to the high spatial resolution and high soft tissue contrast. MRI can identify atrophy in dementia and exclude other diseases like vascular disease, cerebral amyloid angiopathy, brain tumors, and traumatic as well as inflammatory brain changes [1]. Positron emission tomography (PET) with fluorodeoxyglucose ([^18^F]FDG) is however increasingly used to support the clinical diagnosis of patients with suspected dementia, as hypometabolism in certain brain regions can help identify specific types of dementia, including Alzheimer’s disease (AD) and frontotemporal dementia (FTD) [2]. PET has a higher sensitivity for detecting early metabolic changes, which takes place prior to the morphological changes visible on MRI [1]. Hybrid PET/MRI systems have opened up the opportunity for simultaneous PET/MRI acquisitions, enabling fast and convenient examinations for patients with dementia. The information from PET and MRI is complementary, and detection of dementia with the combination of [^18^F]FDG PET and MRI is more accurate than with either of the imaging modalities alone [3].

As a complement to the visual assessment of hypometabolism in PET images performed by nuclear medicine physicians, PET data can be compared to databases of age-matched healthy controls. Z-score maps are then calculated, which represents the number of standard deviations (σ) separating the [^18^F]FDG uptake of the patient and the average of the healthy controls, where moderate hypometabolism is defined as a z-score between − 2 σ and − 3 σ, and severe hypometabolism for a z-score below − 3 σ [4]. A prerequisite for using such quantitative comparisons clinically is quantitatively accurate PET images, which are heavily dependent on attenuation correction (AC). AC is one of the most important corrections that needs to be performed on PET images, but is still challenging when using a PET/MRI system.

For PET/computed tomography (CT) systems, AC is based on CT images (CTAC), which is scaled by a bilinear function to represent the linear attenuation coefficients (LACs) of the 511 keV photons. For PET/MRI systems, alternative methods had to be developed in order to calculate attenuation maps from MRI data since there is no direct relation between the MRI signal and the electron density of tissue. Several proposed brain MRI-based AC (MRAC) methods have demonstrated a small and acceptable bias from CTAC (regional difference within ± 5%) [5]. Most of these promising methods are however not implemented in clinical systems, except for Dixon with bone model that recently became available on the Siemens PET/MRI system (software VE11P). A few studies have compared clinically implemented and research MRAC methods with CTAC in the evaluation of cognitive impairment. Cabello et al. [6] compared Dixon-based (without bone) AC and ultrashort echo time (UTE) AC with four novel MRAC methods. They concluded that Dixon- and UTE-based AC were inferior to the research MRAC methods, both when measuring [^18^F]FDG uptake and z-score accuracy to identify regions with reduced metabolism, compared to CTAC. These findings need to be re-evaluated after the recent software upgrade with modifications to the Dixon and UTE sequences.

The most relevant clinical issue is whether MRAC have an impact on clinical neurodegenerative diagnosis. Werner et al. [7] found that the pattern of hypometabolism remained largely unchanged with Dixon and that the clinical impact was negligible compared to CTAC. Franceschi et al. [8] found similar performance for Dixon and the prototype of Dixon with bone model in visually identifying hypometabolism without z-scores, and also concluded that even Dixon is acceptable for routine clinical evaluation of dementia. Still, the quantitative errors should be further reduced and MRAC methods better imitating CTAC is warranted.

Another factor that potentially can impact the presence of hypometabolism is the choice of reference region. In the comparison to the database of healthy controls, the [^18^F]FDG uptake is normalized to a reference region, which should be unaffected by the disease. The most commonly used reference regions in dementia evaluations are cerebellum and pons, and incorrect AC in these regions can induce a bias in the [^18^F]FDG uptake affecting z-scores throughout the brain. The accuracy of the MRAC methods in the reference region is thus important and should be investigated further.

The aim of this study was to assess the quantitative and clinical impact of the implemented MRAC methods in [^18^F]FDG PET evaluation of dementia (Dixon, Dixon with bone model, UTE) on the Siemens Biograph PET/MRI scanner. Two research MRAC methods (DeepUTE and UCL) presented in the literature were also included for comparison, in addition to CTAC as reference. Secondary aims were to investigate how the choice of reference region influenced the z-scores quantitatively.

## Materials and Methods

### Patients

Twenty-seven consecutive patients with suspected dementia were referred to brain PET/CT and PET/MRI examinations. Nine patients were excluded from this study due to incorrect anatomical position during the PET/CT examination (*n* = 5), misregistration of bone in Dixon_Bone_ MRAC (*n* = 2) (the artifacts could not be removed manually and a new Dixon acquisition was not acquired), aliasing in MRI scans (*n* = 1), and problems with co-registration of PET images to the MNI PET template (*n* = 1). The 18 patients included had a mean age of 69 ± 9 years and a mean weight of 75 ± 16 kg. Patient characteristics and the proposed diagnosis made by a nuclear medicine physician and a neuroradiologist based on PET/CT and MR imaging and clinical referral text is given in Table 1. The study was approved by the Regional Committee for ethics in Medical Research (REC Central) (ref. number: 2013/1371) and all patients gave written informed consent.

**Table 1:** Patient characteristics

Patient	Age (years)	Gender	Proposed diagnosis^a^
1	72	M	Non-specific
2	70	F	FTD
3	49	F	Normal
4	78	F	Normal
5	74	M	FTD
6	64	M	Normal
7	83	F	AD/FTD^b^
8	54	F	Non-specific
9	61	M	Normal
10	82	M	Normal
11	71	F	Non-specific
12	75	F	Normal
13	68	M	Non-specific
14	66	F	Normal
15	72	F	Non-specific
16	63	M	AD
17	69	M	Non-specific^c^
18	70	F	Normal

*AD* Alzheimer’s disease, *FTD* frontotemporal dementia, *Non*-*specific* other subtypes of dementia, and other patterns of hypometabolism that cannot be explained by image artifacts

^a^Diagnosis based on PET/CT + MRI and clinical referral text

^b^Ambiguous clinical information as well as imaging data, but clearly neurodegenerative

^c^Suspicion of normal pressure hydrocephalus (later confirmed clinically and operated with ventricular shunt)

### Image acquisition and reconstruction

Image acquisition was performed on a Biograph mCT PET/CT system (software version VG51C), and subsequently on a Biograph mMR PET/MRI system (software version VE11P) (Siemens Healthcare GmbH, Erlangen, Germany). All patients fasted at least 6 h prior to intravenous injection of [^18^F]FDG (210 ± 46 MBq). The patients were kept blindfolded in a quiet room during the uptake phase prior to the PET/CT examination, which was performed 35 ± 1 min post injection (p.i.), followed by the PET/MRI examination, performed 64 ± 9 min p.i.

Only the low-dose CT scan and the corresponding attenuation map were used (as reference) from the PET/CT examination. The PET (20 min) and MRI (17 min) acquisitions were performed simultaneously, and the MRI protocol consisted of the same sequences as in the clinical MRI protocol for patients with suspicion of dementia (sagittal 3D T1 MPRAGE, coronal T2, transversal FLAIR, GRE T2* (microhemorrhage), and diffusion weighted imaging) in addition to the MRI sequences for MRAC; a high-resolution two-point Dixon VIBE and a UTE sequence. All PET reconstructions were performed on the mMR system using 3D OSEM reconstruction (three iterations and 21 subsets, 344 × 344 image matrix, 4 mm Gaussian filter) and corrections for scatter, randoms, detector normalization, decay, and attenuation.

### Attenuation maps

PET data acquired at the PET/MRI system was reconstructed with the following five MR attenuation maps and a CT attenuation map (presented in Fig. 4) for each patient:

1. Dixon_NoBone_: Implemented at the mMR system. Segmentation-based method that relies on the two-point Dixon VIBE sequence (Brain HiRes), where air, fat, and soft tissue are segmented and assigned predefined discrete LACs (air: 0 cm^−1^, fat: 0.0854 cm^−1^, fat/soft tissue mix: 0.0927 cm^−1^, and soft tissue: 0.1000 cm^−1^).
2. Dixon_Bone_: Implemented at the mMR system (product in the latest software, VE11P). Similar to Dixon_NoBone_, but includes continuous bone information from an integrated bone atlas by registration of MR images of the subject to MR images of the atlas [9, 10]. The atlas contains sets of pre-aligned MR image and bone mask pairs with bone densities as LACs in cm^−1^ at the PET energy level of 511 keV.
3. UTE: Implemented at the mMR system. Segmentation-based method that relies on the two images from the UTE sequence with different echo times (TE_1_ and TE_2_), and segments the image into air (0 cm^−1^), soft tissue (0.1000 cm^−1^), and bone (0.1510 cm^−1^).
4. UCL: Atlas-based method using a database of 41 paired T1-weighted MRI and CT data sets [11–13]. All MRI data sets of the atlas are non-rigidly registered to the patient’s MRI data and normalized correlation coefficients are calculated at each voxel. A pseudo CT is then calculated from averaged weights of the CT data sets based on the correlation coefficients. In this study, T1-weighted MPRAGE was used as input in a web-based tool, after bias correction with FMRIB Software Library (FSL, Oxford Centre for Functional MRI of the Brain, UK), as recommended by the distributor. The returned UCL attenuation map in Houndfield units (HU) was converted to LACs [14] and smoothed with a 4 mm Gaussian filter.
5. DeepUTE: Artificial intelligence approach to MRAC, using a deep learning algorithm [15]. Briefly, the method uses a modified 3D U-net architecture [16] for image-to-image learning of paired UTE and CT data. Compared to [15], the network was here trained using data from 832 adult examinations.
6. CT: Attenuation map generated by converting a low-dose CT scan on the mCT scanner to LACs [14]. The bed and head holder was excluded from the CT attenuation map by making a semi-automatic head mask (CT head mask) with the software MRIcron [17], and the attenuation map was multiplied by 10,000 to get the same order of magnitude as the MRAC maps at the mMR system. The CT attenuation maps did not cover the neck region sufficiently for attenuation correction of the PET data from the PET/MRI system due to differences in the axial field of view of the PET-detectors. The area outside the CT head mask was therefore substituted with the Dixon_Bone_ attenuation map for each patient. The CT image was rigidly registered to the Dixon in-phase image and the same transformation was performed on the CT attenuation map.

The same voxels that were substituted by Dixon_Bone_ in the CT attenuation maps were also substituted by Dixon_Bone_ in all evaluated MR attenuation maps. In order to perform this voxel substitution, the UTE TE_2_ image and the T1w MPRAGE image was registered to the Dixon in-phase image, and the resulting transformations were used on the respective attenuation maps. All registrations were performed with Aliza Medical Imaging 1.35.3 (Bonn, Germany) (using elastix version 4.8 [18, 19]) [20]. To enable import of the modified attenuation maps at the PET/MRI system, all attenuation maps used the header file of Dixon_Bone_ with exchange of the pixel data.

### Quantitative analysis

#### Bone artifacts

After software upgrade (from VB20P to VE11P) of the PET/MRI system, bone artifacts have been observed in the Dixon_Bone_ and UTE attenuation maps. Two of the most severe artifacts seen in the attenuation maps are misplacement of bone segments from other parts of the body found in the Dixon_Bone_ and bone present inside the brain nearby the anterior ventricles in the UTE attenuation maps. The Dixon_Bone_ and UTE attenuation maps were therefore visually inspected for these artifacts.

#### [^18^F]FDG uptake

The [^18^F]FDG uptake in all PET reconstructions were measured in 15 brain regions that were chosen to match the brain regions in the software used for z-score analysis and visual assessment. The regions were in MNI space and taken from the Harvard-Oxford Cortical Structural Atlas, MNI Structural Atlas, and Talairach Daemon Labels in FSL (Oxford Centre for Functional MRI of the Brain, UK). The PET images of the patients were converted to MNI space by co-registration to a dementia-specific [^18^F]FDG-PET template [21, 22]. The PET_DixonBone_ was first registered with elastix to the PET template in a two-step process (rigid and non-rigid registration), and the resulting transform was used on the other five PET images of the same patient for transformation to MNI space. Relative difference (RD) was calculated in each brain region, and was defined as

2 $$ \mathrm{RD}\left(\%\right)=\frac{{\overline{PET}}_{MRAC}-{\overline{PET}}_{CTAC}}{{\overline{PET}}_{CTAC}}\times 100, $$

where ${\overline{PET}}_{MRAC}$ and ${\overline{PET}}_{CTAC}$ is the average activity measured in a brain region in PET_MRAC_ and PET_CTAC_, respectively. The results are presented by using the boxplot function in MATLAB (R2017b). Absolute RDs were also calculated and averaged over patients and brain regions as $\overline{{\mathrm{RD}}_{abs}}$.

#### Z-scores

The visual evaluations were performed with the software Cortex ID (GE Healthcare, Waukesha WI, USA), where z-scores were calculated in 26 brain regions. The database constitutes of 294 healthy controls divided in six age groups, imaged with [^18^F]FDG PET and using a transmission scan of ^68^Ge for attenuation correction. Both cerebellum and pons were used as reference regions in the quantitative analysis. Quantitative comparison of z-scores between PET_MRAC_ (PET_DixonBone_, PET_DixonNoBone_, PET_UTE_, PET_UCL_, PET_DeepUTE_) and PET_CTAC_ were performed by calculating the difference, *D*, and absolute difference, *D*_abs_, in each brain region, where

$$ D={Z}_{MRAC}-{Z}_{CTAC}, and $$

$$ {D}_{abs}=\mid {Z}_{MRAC}-{Z}_{CTAC}\mid, $$

and *D*_*abs*_ was averaged over patients and brain regions as $\overline{D_{abs}}$. The boxplot function in MATLAB was used to present the differences in z-scores.

### Visual evaluation

To limit the number of images in the visual evaluation, MRAC methods were chosen based on the z-score analysis. The best and worst of the clinically implemented MRAC methods were included, in addition to the best research MRAC method. The PET_CT_ was used as reference. Three nuclear medicine physicians (brain PET experience; reader 1: 3 years, reader 2: 10 years, reader 3: 1 year) performed the visual assessments individually. Based on PET images and z-scores, the patients were either categorized as normal or diagnosed with AD, FTD, or non-specific pathology (other subtypes of dementia, like DLB, and other patterns of hypometabolism that cannot be explained by image artifacts). The physicians were blinded for AC method and patient ID, and had no information regarding patient history or MRI.

A second reading was made based on both PET images, z-scores, and MR images by a nuclear medicine physician (reader 3) and a neuroradiologist (4-year experience in neuroradiology and European Diploma in NeuroRadiology (EDiNR)) in conjunction. The best clinically implemented MRAC method based on the z-score analysis was chosen for this second visual evaluation, and PET_CT_ was used as reference. The first and second visual evaluation was done 2 months apart.

PET images and z-scores were evaluated in Cortex ID, while MR images were assessed with the hospitals Picture Archiving and Communication System (PACS; Sectra IDS 7). Cerebellum was chosen as reference region in all visual evaluations.

### Statistical analysis

κ-statistics were calculated (with Stata/MP 15.1, StataCorp LLC, USA) to determine the agreement between PET_CT_ and each PET_MRAC_ in the visual evaluations after correction for the agreement expected by chance. The inter-reader agreement was also calculated for each AC method in the evaluations with three readers. A κ value of 0 indicates no agreement better than chance, and the values were interpreted according to Landis et al. [23] (poor: < 0, slight: 0.00–0.20, fair: 0.21–0.40, moderate: 0.41–0.60, substantial: 0.61–0.80, almost perfect: 0.81–1.00).

## Results

### Quantitative analysis

#### Bone artifacts

**Fig. 1:**
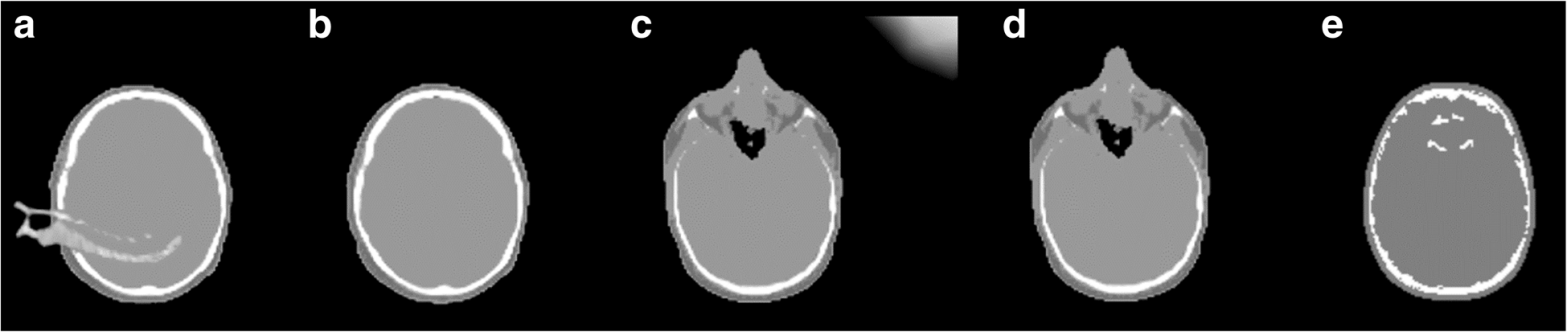
Typical bone artifacts found in attenuation maps from PET/MRI. **a** Dixon_Bone_ attenuation map with large infiltrative bone segment. Attenuation maps like this were only included in the study if the patient had a second acquisition yielding **b** an artifact-free attenuation map. **c** Dixon_Bone_ attenuation map with artifact in the upper right corner, which could be **d** manually removed. **e** UTE attenuation map with smaller bone segments inside the brain nearby the anterior ventricles. These were not removed

Bone artifacts were observed in 22 % (4/18) of the Dixon_Bone_ attenuation maps, while no artifacts were seen in the corresponding Dixon images. New Dixon sequences were acquired for two patients with large bone segments from other parts of the body infiltrating the head (Fig. 1a), resulting in artifact-free attenuation maps (Fig. 1b). Artifacts positioned outside the head (Fig. 1c) were manually removed (Fig. 1d) for the last two patients. Hence, only artifact free Dixon_Bone_ attenuation maps were included in the study. Furthermore, in 89% (16/18) of the UTE attenuation maps, minor bone artifacts were observed inside the brain close to the anterior ventricles (Fig. 1e). The UTE artifacts were not removed.

#### [^18^F]FDG uptake

**Fig. 2:**
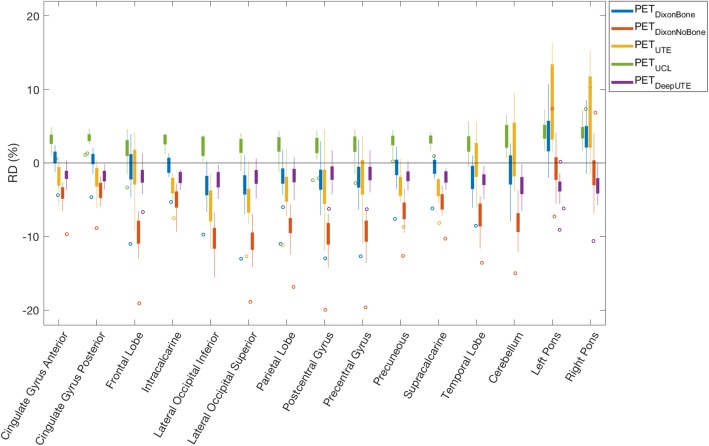
Relative difference in [^18^F]FDG uptake between PET_MRAC_ (PET_DixonBone_, PET_DixonNoBone_, PET_UTE_, PET_UCL_, PET_DeepUTE_) and PET_CTAC_. Top and bottom edges of the boxes indicate 25th and 75th percentiles, while the whiskers extend to the most extreme data points, except for outliers that are marked as circles

The mean absolute relative difference ($\overline{{\mathrm{RD}}_{\mathrm{abs}}}$) in [^18^F]FDG uptake compared to PET_CT_ was the smallest for PET_DeepUTE_ and the largest when omitting bone information in PET_DixonNoBone_ (Table 2). PET_DixonBone_ performed similar to the research MRAC methods, but had slightly larger range of RD. The relative differences in [^18^F]FDG uptake for the different brain regions are presented in Fig. 2. Patient 3, with abnormal anatomy (an arachnoid cyst in the posterior fossa), caused most of the outliers seen in Fig. 2. The attenuation maps with corresponding PET images for all reconstructions are demonstrated for this patient in Additional file 1: Figure S1.

**Table 2:** The mean absolute relative difference ($\overline{RD_{abs}}$) and the range of RD in ^*18*^F[FDG] uptake for the PET_MRAC_ methods compared to PET_CTAC_.

MRAC method	$\overline{{\mathrm{RD}}_{abs}}$ (%) mean ± std	*RD* (%) [min max]
PET_DeepUTE_	2.2 ± 1.5	[− 10.6, 1.7]
PET_UCL_	3.0 ± 1.4	[− 3.3, 7.3]
PET_DixonBone_	2.5 ± 2.4	[− 13.0, 10.7]
PET_DixonNoBone_	7.1 ± 3.7	[− 19.9, 7.4]
PET_UTE_	4.1 ± 3.3	[− 12.7, 16.3]

#### Z-scores

The mean absolute difference ($\overline{D_{\mathrm{abs}}}$) in z-score between CTAC and MRAC was minimized with the research methods (PET_DeepUTE_ and PET_UCL_), which also had the smallest range. Among the clinically implemented methods, PET_DixonBone_ performed best, closely followed by PET_DixonNoBone_. The largest $\overline{D_{\mathrm{abs}}}$ was found with PET_UTE_ (Table 3). For all MRAC methods, smaller differences were found for cerebellum than for pons as reference region.

**Table 3:** The mean absolute difference ($\overline{D_{abs}}$) in z-score between PET_MRAC_ and PET_CT_ and the range of the difference (*D*), with pons and cerebellum as reference regions

MRAC method	$\overline{D_{abs}}$ (σ) (pons) mean ± std	*D* (σ) (pons) [min max]	$\overline{D_{abs}}$ (σ) (cerebellum) mean ± std	*D* (σ) (cerebellum) [min max]
PET_DeepUTE_	0.19 ± 0.16	[− 0.43, 0.76]	0.15 ± 0.11	[− 0.60, 0.60]
PET_UCL_	0.21 ± 0.15	[− 0.69, 0.47]	0.15 ± 0.12	[− 0.64, 0.44]
PET_DixonBone_	0.48 ± 0.27	[− 1.52, 0.31]	0.23 ± 0.20	[− 1.14, 1.42]
PET_DixonNoBone_	0.53 ± 0.35	[− 1.77, 0.74]	0.32 ± 0.28	[− 1.32, 1.67]
PET_UTE_	1.13 ± 0.47	[− 2.85, − 0.02]	0.54 ± 0.40	[− 1.64, 2.49]

**Fig. 3:**
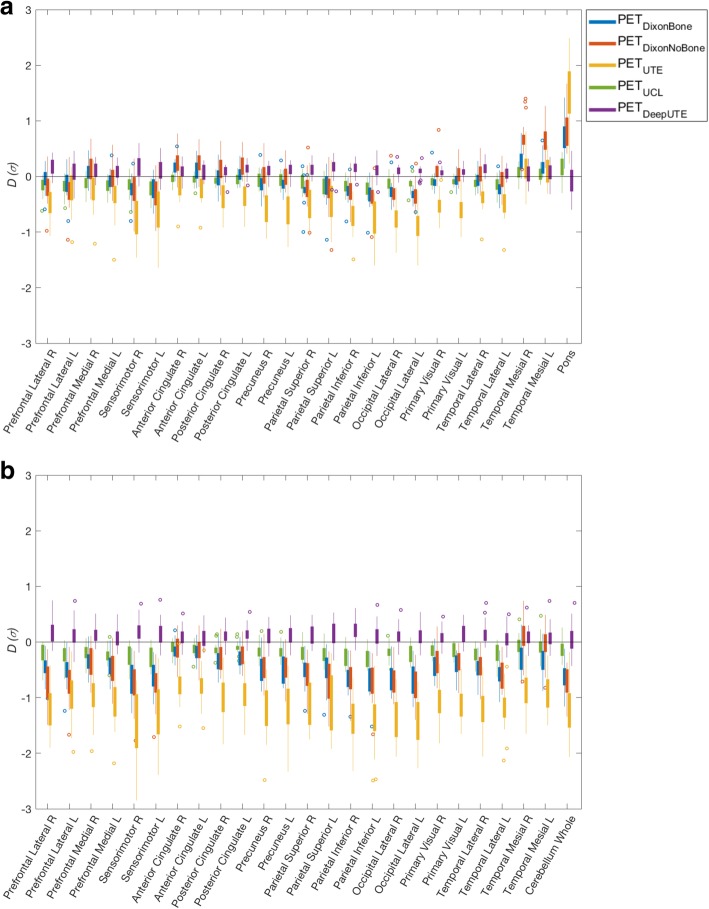
Difference in z-score between PET_MRAC_ (PET_DixonBone_, PET_DixonNoBone_, PET_UTE_, PET_UCL_, PET_DeepUTE_) and PET_CTAC_, for different brain regions, with **a** cerebellum and **b** pons as reference region. Top and bottom edges of the boxes indicate 25th and 75th percentiles, while the whiskers extend to the most extreme data points, except for outliers that are marked as circles

**Fig. 4:**
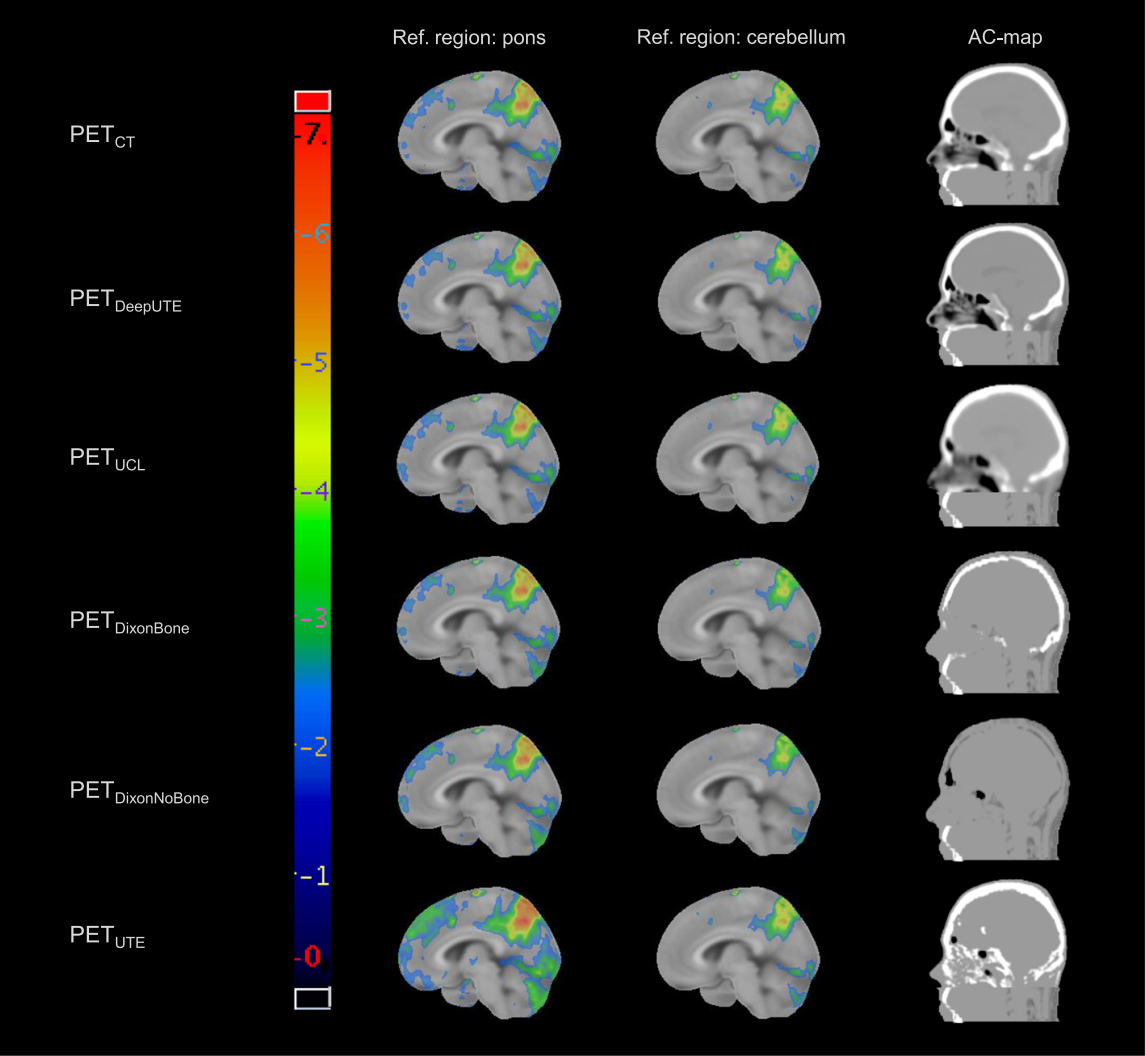
Examples of z-score maps for one patient (number 16) for the included AC methods, with pons and cerebellum as reference regions, and the corresponding attenuation maps

Figure 3 shows that the difference in z-scores between CTAC and the MRAC methods were more stable across brain regions for the research methods than for the clinical methods. PET_DeepUTE_ slightly overestimated and PET_UCL_ slightly underestimated the z-scores compared to PET_CT_ for most brain regions for both reference regions (Fig. 3). The clinical MRAC methods (PET_DixonBone_, PET_DixonNoBone_, and PET_UTE_) yielded lower z-scores than PET_CT_ with pons as reference region, and both over- and underestimated z-scores with cerebellum as reference region (Fig. 3). Examples of z-score maps for one patient with dementia are presented in Fig. 4, where increased hypometabolism is especially pronounced for PET_UTE_ with pons as reference region.

### Visual evaluation

PET_DixonBone_ and PET_UTE_ were chosen for the first visual evaluations (PET only) as these had the best and the worst results of the clinically implemented MRAC methods in the z-score analysis. The research MRAC methods performed relatively equal in the z-score analysis and PET_DeepUTE_ was chosen for the visual evaluation. Furthermore, PET_CT_ was included as reference. The results of the visual evaluations with PET only are presented in Table 4. The agreement in diagnosis between PET_CT_ and PET_DeepUTE_, PET_DixonBone_ and PET_UTE_ was in average for the three readers 74%, 67%, and 70%, respectively (Table 5), and the κ-statistics indicated mostly *moderate* agreement between PET_CT_ and PET_MRAC_. The inter-reader agreement was *fair* for PET_CT_ (κ = 0.30) and *slight* for PET_DeepUTE_ (κ = 0.17), PET_DixonBone_ (κ = 0.19), and PET_UTE_ (κ = 0.10).

**Table 4:** Assigned diagnostic categorization for each patient made by three nuclear medicine physicians from PET images with three MRAC methods (PET_DeepUTE_, PET_DixonBone_, PET_UTE_) and PET_CT_. Cerebellum was used as reference region. Intra-reader discrepancies with PET_CT_ as reference are italicized

Patient	Reader	PET_CT_	PET_DeepUTE_	PET_DixonBone_	PET_UTE_
1	1	N	N	N	N
	2	N	N	N	*NS*
	3	N	N	N	*NS*
2	1	AD	*N*	AD	AD
	2	NS	*FTD*	*FTD*	NS
	3	FTD	*NS*	*NS*	*NS*
3	1	N	N	N	N
	2	N	N	*NS*	*NS*
	3	N	N	N	*NS*
4	1	N	N	*AD*	*AD*
	2	FTD	FTD	FTD	FTD
	3	N	N	N	N
5	1	FTD	FTD	FTD	FTD
	2	AD	*NS*	*NS*	AD
	3	NS	*AD*	*AD*	*AD*
6	1	N	N	N	*AD*
	2	N	N	N	N
	3	N	N	N	N
7	1	AD	*NS*	*NS*	*NS*
	2	AD	AD	*NS*	AD
	3	AD	AD	AD	AD
8	1	AD	*N*	*N*	AD
	2	FTD	FTD	FTD	FTD
	3	N	*NS*	*NS*	*NS*
9	1	N	N	N	*NS*
	2	NS	NS	*N*	NS
	3	NS	*N*	*N*	*N*
10	1	AD	*N*	*N*	AD
	2	N	N	N	N
	3	N	N	N	N
11	1	N	N	N	*AD*
	2	NS	*FTD*	NS	NS
	3	NS	NS	NS	NS
12	1	N	N	N	N
	2	N	N	N	N
	3	N	N	N	N
13	1	N	N	*FTD*	N
	2	NS	NS	NS	NS
	3	NS	NS	NS	NS
14	1	N	N	N	N
	2	N	*FTD*	*FTD*	*FTD*
	3	N	N	*NS*	N
15	1	N	N	N	*AD*
	2	FTD	FTD	FTD	FTD
	3	N	*NS*	*NS*	*NS*
16	1	AD	AD	AD	AD
	2	AD	AD	AD	AD
	3	AD	AD	AD	AD
17	1	AD	AD	*NS*	AD
	2	AD	*NS*	AD	AD
	3	NS	NS	NS	NS
18	1	N	N	N	N
	2	NS	NS	NS	NS
	3	N	N	N	N

*N* normal, *NS* non-specific, *AD* Alzheimer’s disease, *FTD* frontotemporal dementia

**Table 5:** κ-statistics for the agreement between PET_CT_ and PET_MRAC_ (PET_DeepUTE_, PET_DixonBone_, PET_UTE_) for each reader. A κ value of 0 indicates no agreement better than chance, while 1.0 means perfect agreement

	PET_CT_ vs PET_DeepUTE_ Agreement (κ)	PET_CT_ vs PET_DixonBone_ Agreement (κ)	PET_CT_ vs PET_UTE_ Agreement (κ)
Reader 1	77.8% (0.54)	66.7% (0.41)	66.7% (0.47)
Reader 2	72.2% (0.63)	66.7% (0.55)	83.3% (0.78)
Reader 3	72.2% (0.55)	66.7% (0.47)	61.1% (0.39)
Mean of readers	74.1%	66.7%	70.4%

In the second visual evaluation, which also included MRI, PET_DixonBone_ was compared to PET_CT_. When MRI was included in the assessment, the agreement increased to 89% and the *κ*-statistics indicated *almost perfect* agreement (*κ* = 0.82) (Table 6) according to Landis et al. [23].

**Table 6:** Assigned diagnostic categorization made by one nuclear medicine physician (reader 3) and one neuroradiologist in conjunction for PET (PET_CT_, PET_DixonBone_) and MRI. Discrepancies from PET_CT_ + MRI are italicized

Patient	PET_CT_ + MRI	PET_DixonBone_ + MRI
1	NS	NS
2	NS	NS
3	N	N
4	N	N
5	FTD	FTD
6	N	N
7	FTD	*AD* ^a^
8	N	N
9	N	N
10	N	N
11	Ns	NS
12	N	N
13	NS	NS
14	N	N
15	NS	NS
16	AD	*NS*
17	NS ^b^	NS^b^
18	N	N

*AD* Alzheimer’s disease, *FTD* frontotemporal dementia

^a^Defined as both FTD and AD in the proposed diagnosis based on PET/CT and MR imaging and clinical referral text (Table 1)

^b^Suspicion of normal pressure hydrocephalus

## Discussion

The impact of MRAC on dementia assessment was evaluated in this study by comparing [^18^F]FDG uptake, z-scores, and clinical interpretation between PET_MRAC_ and PET_CT_. The absolute mean quantitative differences in z-scores were small relative to the definition of hypometabolism for most MRAC methods with cerebellum as reference region, and especially for the research methods. Interpretation with PET alone yielded high uncertainties, while assessment with both PET and MRI resulted in almost perfect agreement between PET_CT_ and PET_DixonBone_.

The bone artifacts found in the clinically available MRAC methods highlights the need for careful inspection of the attenuation maps in all brain examinations. In the Dixon_Bone_ attenuation maps, the artifacts were caused by misregistration between the Dixon images and the bone-template, misplacing large bone segments from other parts of the body in the brain. Due to the severity of these artifacts, they were removed by either acquiring a new Dixon acquisition free of this artifact, or manually when found outside the brain. Although not evaluated quantitatively, this artifact would likely induce large errors in the attenuation corrected PET images. The minor bone artifacts observed in most UTE attenuation maps were caused by changes in the UTE sequence and/or attenuation map algorithm after the software upgrade, and persisted even after acquiring new UTE images. Since the UTE attenuation maps are used clinically, they were not excluded from the current study. In clinical routine, a reliable and stable MRAC method is crucial and these problems need to be solved. The attenuation map errors have been reported to Siemens Healthcare, and will hopefully be solved in the near future. In the meantime, some of the artifacts can be avoided by implementing better procedures among radiographers to detect the artifacts and acquire new MR-based attenuation maps in such cases before the patient leaves the scanner table.

The research MRAC methods, as well as PET_DixonBone_, all demonstrated small absolute differences compared to PET_CT_ regarding [^18^F]FDG uptake, although the research methods had smaller RD range. Some outliers were observed in the analysis, and most of them were caused by a patient with an abnormal anatomy (arachnoid cyst in posterior fossa). DeepUTE gave least outliers for this patient with abnormal anatomy (2/15 brain regions), while Dixon_Bone_ and Dixon_NoBone_ yielded most outliers for this patient (10/15 brain regions). For absolute differences in [^18^F]FDG uptake, the trend was the same as in previous studies [5–7, 24], with descending performance for PET_UCL_, PET_DixonBone_, PET_UTE_, and PET_DixonNobone_ (DeepUTE has not been included in previous studies). PET_UTE_ yielded particularly large variations in the pons, probably due to misclassification of bone in that region, which makes pons not suited as a reference region with UTE AC. Furthermore, we found that the LACs for soft tissue were slightly higher with UCL AC and slightly lower with DeepUTE AC compared to the CTAC, which probably caused the general over- and underestimation of [^18^F]FDG uptake for the two methods, respectively.

For the z-score evaluation, the research MRAC methods yielded the best performance and the differences in z-scores between PET_MRAC_ and PET_CT_ were generally small compared to the definition of hypometabolism, except for PET_UTE_, when using cerebellum as reference region. Of note, PET_DixonBone_ and PET_DixonNoBone_ yielded similar results for the z-scores, indicating that the missing bone information did not have a remarkable impact on z-scores. Despite small average differences in z-scores to PET_CT_ for most MRAC methods with cerebellum as reference region, large outliers were present for the clinical MRAC methods with a deviation from PET_CT_ > 1 σ, which can have a considerable impact on a z-score assessment. Hence, it is highly desirable to implement the research methods at the clinical PET/MRI systems as soon as possible to avoid large biases in the z-score assessment.

Since the calculation of z-scores use a reference region for normalization, the accuracy of AC in the reference region is of particular importance as bias in this region will affect the hypometabolism globally. Available reference regions in the software used for visual evaluation in the current study were pons, cerebellum, and global cerebral cortex. The extent of hypometabolism can however be underestimated with global normalization [25]. Therefore, only cerebellum and pons were used in this study, and differences in z-scores between MRAC and CTAC were found to be smaller with cerebellum than pons as reference region for all MRAC methods. Although glucose metabolism in the pons have been found to be least affected by dementia among several reference regions [26], the small size makes this region prone to bias and the surrounding inhomogeneous bone affects both attenuation and scatter [25]. Cerebellum is larger and less prone to bias, and the cerebellar glucose metabolism is not significantly reduced for AD patients, except for severe AD [25, 27].

Based on our results, Dixon_Bone_ with cerebellum as reference should be preferred among the clinically implemented MRAC methods when assessing z-scores. However, for patients with abnormal anatomy and/or unusual tissue density, atlas-based methods should be used with caution [28]. In these cases, or when bone artifacts are present, Dixon_NoBone_ could probably be used as an alternative for z-score assessment in the evaluation of dementia.

The visual evaluations with PET only yielded moderate agreement between PET_CT_ and PET_MRAC_ in general. Highest agreement was found for PET_DeepUTE_, but the other MRAC methods performed quite similarly. Least false positive errors were found for PET_DeepUTE_ compared to PET_CT_, while false negative errors were highest for PET_DeepUTE_ and PET_DixonBone_. These results seem to be in agreement with the quantitative results, as PET_UTE_ underestimated z-scores, inducing false positive errors, while PET_DeepUTE_ slightly overestimated z-scores and tends to more often change pathology to normal than the opposite. However, the inter-reader agreement was low, which indicates that the visual assessment of [^18^F]FDG PET in dementia is difficult and subjective, and that these evaluations were influenced by additional factors than the different AC methods. Another study by Werner et al. [7], evaluating the clinical impact of different AC methods, demonstrated a higher agreement between the readers; however, the categorization of diagnosis was not the same as in our study, which could have caused less discrepancy in their results.

Due to the large discrepancies in the PET only evaluations, another assessment including MRI was performed. Adding MRI information yielded almost perfect agreement between MRAC and CTAC readings according to the *κ*-statistics, and in the two cases of discrepancies between PET_CT_ + MRI and PET_DixonBone_ + MRI, the discrepancies were due to different subtypes of dementia. The improvement by including MRI was probably due to the ability to discard areas of hypometabolism due to other pathologies and normal variants (e.g., age-related atrophy, enlarged ventricles, and mega cisterna magna). Furthermore, information of neurodegenerative processes such as hippocampal atrophy (as seen in AD), focal cortical atrophy (as seen in FTD), and white matter hyperintensities (as seen in microvascular disease) was important complementary information to the PET findings. In a clinical setting with all clinical information and imaging available, the discrepancies between MRAC and CTAC would probably be further decreased, but this should be verified in studies with larger patient cohorts.

A limitation of this study is the small number of patients, and hence few patients having dementia and low diversity in diagnoses and severity. Furthermore, PET images suffer from partial volume effects due to the limited resolution that cause spill-out from one region to another. This was not corrected for and could cause a significant effect on hypometabolism from normal aging [29]. However, the aim of this study was to compare MRAC and CTAC, and not the exact diagnosis. Another factor that may affect the z-scores is that the PET images in the database of Cortex ID were acquired and reconstructed differently than the PET images in this study. Still, the relative differences between CTAC and MRAC should be unaffected.

## Conclusion

The quantitative differences in z-scores between CTAC and most MRAC methods were small relative to the definition of hypometabolism, with cerebellum as reference region. Although the research MRAC methods performed slightly better than the clinically implemented MRAC methods regarding calculations of the z-scores, the visual evaluations with PET and MRI demonstrated almost perfect agreement between Dixon_Bone_ and CTAC. Our results indicate that Dixon_Bone_ with cerebellum as reference region should be preferred among the clinically implemented MRAC methods when using Siemens PET/MRI system for dementia assessment with [^18^F]FDG PET/MRI. Although, inspection of the attenuation maps is a prerequisite for the use of PET/MRI in dementia evaluation.

## Additional file

Additional file 1: **Figure S1.** Attenuation maps (top row) with corresponding PET images (bottom row) for patient number 3 with abnormal anatomy. (a) CT, (b) UCL, (c) DeepUTE, (d) Dixon_Bone_, (e) Dixon_NoBone_ and (f) UTE. (PNG 1967 kb)

## Data Availability

The datasets used and analyzed during the current study are available from the corresponding author on reasonable request.

## Abbreviations

AC: Attenuation correction
AD: Alzheimer’s disease
CT: Computed tomography
CTAC: CT-based AC
[^18^F]FDG: Fluorodeoxyglucose
FTD: Frontotemporal dementia
HU: Houndfield units
LACs: Linear attenuation coefficients
MRI: Magnetic resonance imaging
MRAC: MRI-based AC
PET: Positron emission tomography
RD: Relative difference
UTE: Ultrashort echo time
