# Supplementary figures and images for: Quantitative and clinical impact of MRI-based attenuation correction methods in [18F]FDG evaluation of dementia
Source: EJNMMI Res. 2019 Aug 24;9:83. doi: 10.1186/s13550-019-0553-2 (PMC6708519; doi:10.1186/s13550-019-0553-2)

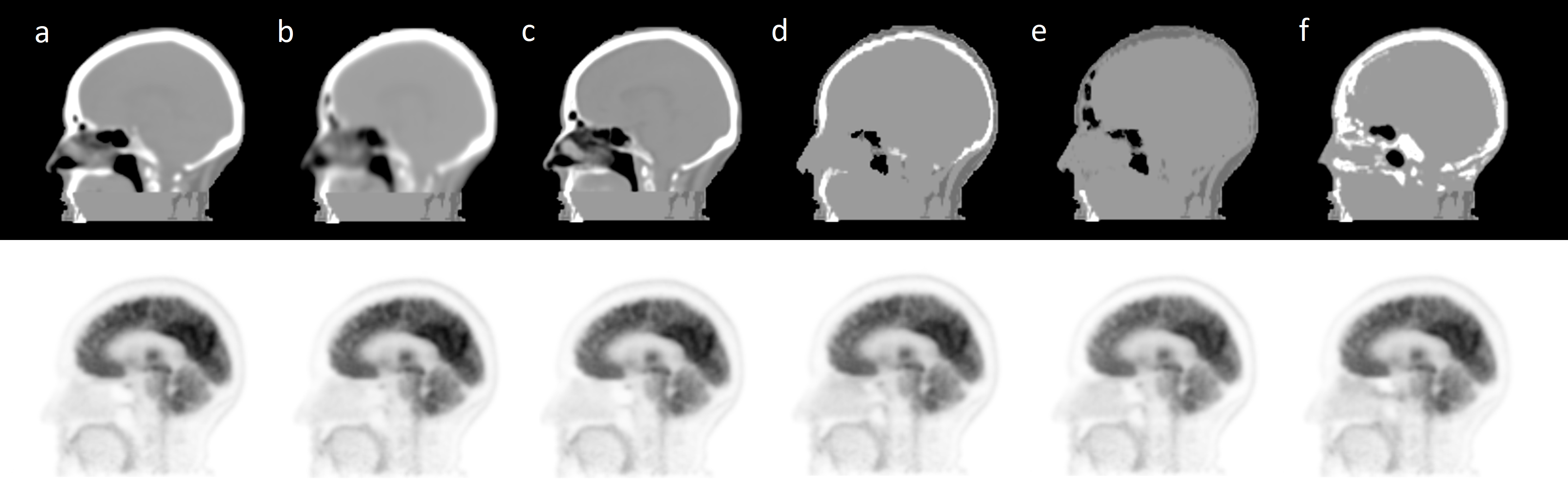

Supplement: Supplementary file 1 — Figure S1. Attenuation maps (top row) with corresponding PET images (bottom row) for patient number 3 with abnormal anatomy. (a) CT, (b) UCL, (c) DeepUTE, (d) DixonBone, (e) DixonNoBone and (f) UTE. (PNG 1967 kb) [file 13550_2019_553_MOESM1_ESM.png]
